# Multiplex PCR assay for identification of commonly used disarmed *Agrobacterium tumefaciens* strains

**DOI:** 10.1186/2193-1801-3-358

**Published:** 2014-07-15

**Authors:** Farah Deeba, Muhammad Zeeshan Hyder, Shahzad Hussain Shah, Syed Muhammad Saqlan Naqvi

**Affiliations:** Department of Biochemistry, PMAS Arid Agriculture University Rawalpindi, Murree Road, 46300 Rawalpindi, Pakistan; Department of Biosciences, COMSATS Institute of Information Technology, Islamabad, Pakistan

**Keywords:** Ach5FtsZ, *Agrobacterium tumefaciens*, C58GlyA, Multiplex PCR, Plant transformation, pTiBo542

## Abstract

The success of Agrobacterium mediated plant transformation depends to a certain extent on appropriate selection of the *A. tumefaciens* strain for a particular plant species. Many stages in a plant transformation procedure are prone to bacterial contamination with similar antibiotic resistance that may compromise the identity of the *A. tumefaciens* strain used, in turn adversely affecting success of a transformation experiment. Different primer sets were designed to exploit genetic differences among different strains of *A. tumefaciens* which are commonly used for plant genetic transformation, to identity confirmation as well as to distinguish them from one another. The primer sets Ach5FtsZ-F/R specific for Ach5 and C58GlyA-F/R specific for C58 were designed on chromosomal DNA while primer sets pTiBo542-F/R and nptI-F/R specific for plasmid pTiBo542 are capable to identify and distinguish these strains from one another. These primer sets when used simultaneously in multiplex PCR, produce a pattern which uniquely identifies all these strains and distinguishes them except for GV3101 and C58C1, which can further be distinguished from each other by rifampicin screening. The multiplex PCR assay and primers being reported here serve as a valuable tool in determining the identity of *A. tumefaciens* strains at any stage of plant transformation procedure.

**Electronic supplementary material:**

The online version of this article (doi:10.1186/2193-1801-3-358) contains supplementary material, which is available to authorized users.

## Background

*Agrobacterium tumefaciens*-mediated transformation is the most efficient and widely employed method for the production of transgenic plants. *A. tumefaciens* is a gram negative, rod shaped, aerobic, soil phytopathogen that belongs to genus *Agrobacterium* in family *Rhizobiaceae* (Rhouma et al. 2006). In nature *A. tumefaciens* genetically transforms host plants and causes crown gall tumors at wounded sites (Smith and Townsend 1907). The *Agrobacterium*-plant cell interaction is the only natural example of trans-kingdom gene transfer. Nevertheless, one of the obstacles in using *A. tumefaciens* for desired gene transfer was presence of genes for plant growth regulators on T-DNA of naturally occurring strains such as Chry5 and A281 (Hood et al. 1987) which after integration into the host genome results in crown gall formation. To circumvent this problem, a number of disarmed *A. tumefaciens* strains containing the non-oncogenic *vir* helper plasmids has been developed including LBA4404 (Hoekema et al. 1983), GV3101 (Holsters et al. 1980), C58C1 (Deblaere et al. 1985), EHA101 (Hood et al. 1986) and EHA105 (Hood et al. 1993) which are routinely being used in plant transformation experiments around the world and show variable efficiencies in transforming different types of plants.

*A. tumefaciens* can transfer DNA to broad group of organisms including dicot and monocot angiosperm species (Porter 1991; Vaudequin-Dransart et al. 1995) and gymnosperms (Wenck et al. 1999). *A. tumefaciens* can also transform yeast (Bundock et al. 1995), fungi (Bundock and Hooykaas 1996) and human cells (Kunik et al. 2001). There are many advantages of *A. tumefaciens* mediated transformation over direct transformation methods such as Polyethylene glycol (PEG) transfer, microinjection, protoplast and intact cell electroporation and microprojectile bombardment. These include efficiency, integration of small copy number of T-DNA into plant chromosomes and stable expression of transferred genes (Koncz et al. 1989; Olhoft et al. 2004). Genetically modified plants are generally fertile and the foreign genes are often transmitted to progeny in a Mendelian manner (Rhodora and Thomas 1996).

Agrobacterium-mediated transformation is a multifaceted interaction and *A. tumefaciens* strain plays major role in transformation efficiency. A number of factors, such as plant genotype, explant types, agrobacterium strains, selection marker genes and various tissue culture conditions are critical for transformation. Therefore, optimization of such factors is of significant importance for the establishment of successful transformation systems in plants. Different Agrobacterium strains are used to optimize T-DNA delivery into host plant genome (Subramanyam et al. 2011; Kim et al. 2013) because Different *A. tumefaciens* strains have different chromosomal backgrounds and may affect the range of plants susceptible to T-DNA transfer (Hood et al. 1993).

A typical plant transformation protocol using *A. tumefaciens* requires revival of Agrobacterium stock on some solid medium with appropriate antibiotic added, then growth in liquid medium to prepare competent cells for electrical or chemical competency to enable them for uptake of plasmid DNA followed by selection of transformed colonies on solid medium. The selected colonies are then grown in liquid medium for use in co-infection process (Shrawat et al. 2007). These multiple steps of growth on solid and liquid media are the stages where culture of original *A. tumefaciens* cells are prone to contamination by other Agrobacterium strains used in the lab or by other bacteria with similar antibiotic resistance pattern. Therefore it is very important for the successful transformation to ensure that the culture being used is of desired strain of *A. tumefaciens*.

Currently naturally occurring pathogenic *Agrobacterium* are identified by serological techniques (Cubero et al. 1999), physiological tests Michel et al. 1990), biochemical methods (Holmes and Roberts 1981), immunological methods (Nesme et al. 1992; Shirasu et al. 1994) and DNA hybridization tests (Furukawa et al. 1984) which are time consuming and costly. In addition PCR based rapid identification systems have also been devised for universal identification of naturally occurring *Agrobacterium* species and *A. tumefaciens* strains (Haas et al. 1995; Sawada et al. 1995; Cubero et al. 1999). However no method is available which can be used to distinguishably identify different disarmed strains of *A. tumefaciens* used for plant transformation purposes. Therefore, the present study was aimed to develop a rapid multiplex PCR assay which can identify as well as distinguish commonly used disarmed *A. tumefaciens* strains employed for plant transformation.

## Results

**Figure 1:**
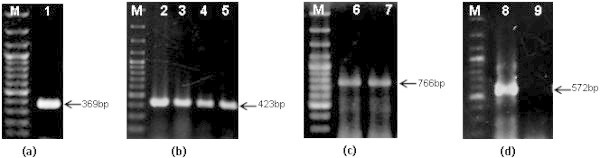
**PCR amplification of different** ***A. tumefaciens*** **strains using various primer sets developed in the study. (a)** from genomic DNA template of LBA4404 strain using Ach5FtsZ-F/R primer set (Lane 1). **(b)** from genomic DNA templates of GV3101, C58C1, EHA101 and EHA105 strains respectively using C58GlyA-F/R primer pair (Lanes 2–5). **(c)** from EHA101 and EHA105 plasmid DNA templates respectively using pTiBo542-F/R primer set. Lane 6, 7). **(d)** from EHA101 genomic and EHA105 plasmid DNA templates respectively using nptI-F/R primer pair (Lane 8, 9). In all panels first lane marked M represents O’RangeRuler™ 100 bp DNA Ladder (Fermentas Lithuania UAB).

The primer set Ach5FtsZ-F/R produced expected specific band of 369 bp (Figure 1a) by PCR of total genomic DNA isolated from LBA4404 strain, while no amplification was observed in PCR of rest of the *A. tumefaciens, A. vitis*, *A. rhizogenes* strains (Additional file 1: Figure S1a). Hence this primer set uniquely identified and distinguished LBA4404 from rest of the Agrobacterium strains used in this study. The primer set C58GlyA-F/R identified GV3101, C58C1, EHA101 and EHA105 strains by amplifying a specific band of about 423 bp from genomic DNA of these strains (Figure 1b) but showed no amplification with DNA of other strains (Additional file 1: Figure S1b). Similarly the primer set pTiBo542-F/R identified EHA101 and EHA105 strains by uniquely producing expected specific band of 766 bp (Figure 1c) from plasmid DNA template of both the strains and also distinguished these two strains from GV3101, C58C1 and LBA4404. This primer set, too, showed no cross-reactivity with any other Agrobacterium strains used (Additional file 1: Figure S1c). The primer set nptI-F/R amplified expected specific band of 572 bp (Figure 1d) only from plasmid DNA template of EHA101 and showed no amplification from plasmid of EHA105 and other strains (Additional file 1: Figure S1d) hence serves to distinguish these strains from one another. The pattern of amplification of these primer sets with various strains used is summarized in Table 1. The specificity of amplification was confirmed by sequencing of these amplified products (data not shown). The results indicated that all the primer sets specifically amplify targeted regions.

**Table 1:** **The reactivity of various primer sets in multiplex PCR amplification to differentiate different Agrobacterium and** ***E. coli*** **strains**

Primers pair set	Tm (°C) Used		***Agrobacterium***species								***E. coli***strains	
			***Agrobacterium tumefaciens***					***Agrobacterium vitis***		***Agrobacterium rhizogenes***		
		Strains	GV3101	C58C1	EHA105	EHA101	LBA4404	Ag57	Ag63	R3	DH5α	BL21
C58GlyA-F/R	61		+*	**+**	**+**	**+**	-*	**-**	**-**	-	**-**	**-**
pTiBo542-F/R	61		**-**	**-**	**+**	**+**	**-**	**-**	**-**	**-**	**-**	**-**
nptI-F/R	61		**-**	**-**	**-**	**+**	**-**	**-**	**-**	**-**	**-**	**-**
Ach5FtsZ-F/R	61		**-**	**-**	**-**	**-**	**+**	**-**	**-**	**-**	**-**	**-**

*The reactivity of primer set with various Agrobacterium strains is indicated with (+) sign in case of positive PCR amplification while with (-) sign in case of no amplification.

**Figure 2:**
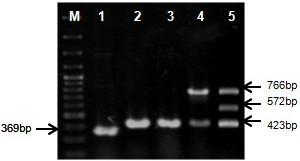
**Multiplex PCR and specificity assessment of C58GlyA-F/R, Ach5FtsZ-F/R, pTiBo542-F/R and nptI-F/R primer pairs for various** ***A. tumefaciens*** **strains.** Multiplex PCR using combined primer sets to amplify total genomic DNA of LBA4404, GV3101, C58C1, EHA105 and EHA101 strains respectively (lanes 1–5). All the strains produced specific bands at 61°C and showed no incompatibility or false priming. M is O’RangeRuler™ 100 bp DNA Ladder (Fermentas Lithuania UAB).

These primer sets after individual optimization, were subjected to multiplex PCR reaction (containing all the four primer sets) so that a single reaction can be used to identify and distinguish different *A. tumefaciens* strains. The multiplex PCR with combined primer sets at 61°C with LBA4404, GV3101, C58C1 EHA105 and EHA101 DNA templates (Figure 2) served in identification by amplifying specific bands of expected sizes i.e. a 369 bp band from LBA4404, a 423 bp band each from GV3101, C58C1, EHA101 and EHA105, while a 766 bp band from EHA101 and EHA105 strains and a 572 bp band only from EHA101. There was no amplification from *E. coli* strains with multiplex primers (Additional file 2: Figure S2) which indicate that these primer sets uniquely identified and distinguished disarmed *A. tumefaciens* strains used in this study. The absence of any non-specific band in multiplex PCR indicated compatibility among primers for combined usage in a single reaction without any interference among one another.

The multiplexing also produced a banding pattern which uniquely distinguishes among LBA4404, EHA105, EHA101, GV3101 and C58C1 strains from one other. The discrimination between GV3101 and C58C1 could not be achieved by these primers, though once identified with C58GlyA-F/R primer set these two strains can be distinguished by selection on rifampicin to which GV3101 strain is resistant.

## Discussion

Agrobacterium-mediated transformation aimed at improving quality and productivity of economically important crops such as wheat, rice, sugarcane, cotton, canola, tomatoes and potatoes as well as of a growing list of other important crops and forest plant species, is a routine in many labs around the globe (Arif et al. 2009). It is also a very important technique used in the quest to understand the biology of host-microbe interaction. Agrobacterium strains which differ in their efficiency to transform various plant species owing to genetic differences in their chromosomal DNA and/or Ti plasmid, have been developed (Kikuchi et al. 2005; Wang et al. 2007).

The success of plant genetic transformation experiment depends on many factors, one of which is appropriate selection of the strain for a particular plant species. For this purpose, different disarmed *A. tumefaciens* strains are used to optimize the transformation of a particular plant. Subramanyam et al. (2011) did banana transformation by using *A. tumefaciens* strains LBA4404, EHA101 and EHA105. He found EHA105 as most effective strain in comparison to LBA4404 and EHA101. Similarly, Kim et al. (2013) used three *A. tumefaciens* strains (LBA4404, EHA105, and GV3101) for reed transformation and obtained high transformation efficiency with EHA105 as compared to LBA4404 and GV3101. This difference might be due to the different virulence (vir) regions and different chromosomal backgrounds of the strains (Hellens et al. 2000) and interaction between agrobacterium and host plant components.

Currently the most commonly employed method for distinguishing various Agrobacterium strains is the use of selective antibiotics against which, certain strains are resistant. A particular strain is maintained on selective antibiotic throughout different steps in transformation procedure including preservation in stock cultures, transformation of the strain with certain recombinant vector carrying gene of interest and co-culture with target plant. These steps require repeated growth of a particular strain on solid and liquid culture media, which are potential phases for contamination of the culture with other microbes harboring resistance against selective antibiotic used. In such conditions the chances of a successful experiment reduces dramatically which can be avoided if the identity of a strain could be established during different experimental steps or whenever needed.

In this study the multiplex PCR assay was developed by designing primer sets which can distinguish different commonly used *A. tumefaciens* strains based on genetic differences in their chromosomal and Ti plasmid DNA. The LBA4404 strain has Ach5 chromosomal background while GV3101, C58C1, EHA101 and EHA105 have C58 chromosomal background. The sequence of whole C58 chromosome is available in GenBank (Goodner et al. 2001) while sequence of only seven genes from Ach5 chromosome were found in public domain (Marri et al. 2008). The sequence comparison of these genes with C58 chromosomal sequence showed FtsZ gene to have appropriate sequence diversity suitable for primer designing. FtsZ gene is a GTPase that is similar to eukaryotic tubulin and is essential for cell division in bacteria (Beall and Lutkenhaus 1991). The forward and reverse primers in Ach5FtsZ-F/R set were designed in such a way that their 3′ends could only amplify FtsZ gene from Ach5 chromosome background but not from C58 chromosome. This primer set therefore served to identify LBA4404 by amplifying a band from its genomic DNA and uniquely distinguishes it from GV3101, C58C1, EHA101 and EHA105 strains which show no amplification. Similarly, the identification of GV3101, C58C1, EHA101 and EHA105 was made by designing C58GlyA-F/R primer set in such a way that the 3′ends of forward and reverse primers could only amplify GlyA gene from C58 chromosomal background but not from Ach5 chromosome. GlyA gene encodes serine hydroxymethyltransferase which is responsible for interconversion of serine, glycine, and one-carbon (C1) units and is the cell’s major source of C1 units (Mudd and Cantoni 1964). The primer set C58GlyA-F/R identifies GV3101, C58C1, EHA101 and EHA105 strains by amplifying GlyA gene only from C58 chromosome and hence discriminates between these strains and LBA4404.

The EHA101 and EHA105 strains contain plasmid pTiBo542 which is not present in GV3101 and C58C1 strains and serves as a mean to genetically discriminate among these strains. The primer set pTiBo542-F/R was designed on accF gene which encodes an enzyme involved in catabolism of opines (Kim and Farrand 1997). The primer set amplifies a region of accF gene present on Ti plasmid pTiBo542 from both EHA101 and EHA105 and distinguishes them from rest of the strains (Figure 2) which show no amplification. Similarly, primer set nptI-F/R was designed on nptI gene which encodes neomycin phosphotransferase enzyme responsible for inactivation of kanamycin antibiotic by phosphorylation. This primer set distinguishes EHA101 which harbors kanamycin resistance from EHA105 and rest of the strains.

The limited knowledge available about the genome sequences of C58C1 and GV3101, did not allow developing PCR primers to discriminate C58C1 from GV3101. The GV3101 is a spontaneous mutant of C58C1 strain and shows resistance to rifampicin (Deblaere et al. 1985). The exact genetic differences in DNA sequences of both the strains are not yet determined. Therefore the current assay identifies both the strains simultaneously and distinguishes them from rest of the strains but the distinction between C58C1 and GV3101 can only be achieved by using rifampicin as selective antibiotic to which GV3101 strain is resistant.

Multiplex PCR reaction worked efficiently for LBA4404, GV3101, C58C1, EHA105 and EHA101. Amplification of specific bands was observed which show that these primer sets are compatible to one another during simultaneous PCR amplification reaction and this allowed us to distinguish desired *A. tumefaciens* strains from the given one in a single PCR.

## Conclusion

Conclusively, the multiplex PCR assay developed in this study is a valuable tool capable of helping in monitoring the purity and identification of some commonly used *A. tumefaciens* strains for plant transformation. This robust assay is quick, sensitive and specific enough to be applied at any stage during the transformation process.

## Materials and methods

Five disarmed strains of *A. tumefaciens* commonly used for plant genetic transformation including GV3101 (pGreen and co-resident pSoup; http://www.pgreen.ac.uk), LBA4404 (pAL4404), C58C1 (cured), EHA101 (pEHA 101(pTiBo542ΔT-DNA)) and EHA105 (pEHA 105(pTiBo542ΔT-DNA)), as well as one wild type strain (At10), one *A. rhizogenes* strain (R3), two *A. vitis* strains (Ag57, Ag63) and two *E. coli* strains (DH5α, BL21) were used in this study. All these strains were maintained on agar plates and in glycerol stocks containing LB (Luria-Bertani) medium except for EHA101 and EHA105 for which YEP (Yeast-Extract Peptone) medium was used. Media were supplemented with 10 mg/L, 20 mg/L and 50 mg/L rifampicin for EHA101 and EHA105, At10 and GV3101 strains respectively, while LBA4404 was maintained on media containing 50 mg/L of both rifampicin and streptomycin.

Total genomic DNA was isolated using protocol of Channarayappa (2006). Briefly, 1.5 mL aliquot from a 3 mL over-night grown culture was centrifuged at 15,500 g for 3 minutes. The resultant pellet was resuspended in 200 μL of lysis buffer (40 mM Trizma base, 20 mM sodium acetate, 1 mM EDTA, 1% SDS, pH 7.8 adjusted with acetic acid) followed by the addition of 66 μL of 5 M NaCl, The lysate was centrifuged at 15,500 g for 10 minutes. Clear supernatant was collected in a fresh tube and treated with 1 μL of RNaseA (10 mg/mL) following incubation at 37°C for 30 minutes. After incubation the lysate was extracted with equal volume of chloroform/isoamyl alcohol (24:1) and centrifuged at 15,500 g for 6 minutes. DNA was precipitated by adding 2.5 volumes of ice cold ethanol (95%) and kept at -20°C for 30 minutes before centrifugation at 15,500 g for 6 minutes. The resultant pellet was washed with 0.5 mL of ice cold 70% ethanol, dried and dissolved in 50 μL of TE buffer (10 mM Trizma base, 1 mM EDTA) for an initial storage at 4°C for one night and then at -20°C until used. Plasmid DNA was isolated using miniprep protocol according to Sambrook and Russel (2001).

Primers were based on genomic DNA sequences of different *A. tumefaciens* strains publically available in GenBank. The accession numbers and regions amplified with respective primer sets are given in Table 2. The propensity of a primer to make homo or hetero duplex was analyzed by OligoIDT Analyzer (http://eu.idtdna.com/analyzer/applications/oligoanalyzer/default.aspx). Designed primers were also screened against nucleotide database in GenBank by BLASTN (Altschul et al. 1997) program for any similarity with *Rhizobium* strains being closely related to *A. tumefaciens* strains. These primers showed no homology to nucleotide sequence of any other *Agrobacterium* or *Rhizobium* strain.

**Table 2:** **Strategy for Primer Designing**

***A. tumefaciens***Strain	Chromosome	Antibiotic gene on chromosome	Ti Plasmid	Antibiotic gene on plasmid	Opine	Target gene (Accession Number) (Nucleotide coordinates)	Primers name and sequence	Primer Tm (°C)	Product size (bp)	References
GV3101	C58	Rifampicin	Cured	-	Nopaline	GlyA gene (Accession no. **EU334236.1**) (1160791-1161213 nt)	C58 GlyA-F: 5′- CCACCACCACGACGCACAAGTCT -3′ C58 GlyA-R: 5′-TGCCGAGACGGACACCCGAC -3′	63.7 64.3	423	(Holsters et al. 1980)
C58C1	C58	-	Cured	-	Nopaline					(Deblaere et al. 1985)
EHA101	C58	Rifampicin	pEHA 101(pTiBo542ΔT-DNA)	Kanamycin	Succinamopine					(Hood et al. 1986)
EHA105	C58	Rifampicin	pEHA 105(pTiBo542ΔT-DNA)	-	Succinamopine					(Hood et al. 1993)
EHA101	C58	Rifampicin	pEHA 101(pTiBo542ΔT-DNA)	Kanamycin	Succinamopine	accF gene (Accession no. **NC_010929**) (237186-237952 nt)	pTiBo542-F: 5′-CCCGCTGAGAATGACGCCAA-3′ pTiBo452-R: 5′-CCTGCGACACATCGTTGCTGA-3′	60.4 60.2	766	(Hood et al. 1986)
EHA105	C58	Rifampicin	pEHA 101 (pTiBo542ΔT-DNA)	-	Succinamopine					(Hood et al. 1993)
EHA101	C58	Rifampicin	pEHA 101(pTiBo542ΔT-DNA)	Kanamycin	Succinamopine	nptI gene (Accession no. **D85525.1**) (929-357 nt)	nptI-F: 5′-CTGCGATTCCGACTCGTCCA-3′ nptI-R: 5′-CGGGCAATCAGGTGCGACA-3′	59.4 59.5	572	(Hood et al. 1986)
LBA4404	Ach5	Rifampicin	pAL4404	Spectinomycin & streptomycin	Octopine	FtsZ gene (Accession no. **EU334225.1**) (48-416 nt)	Ach5FtsZ-F: 5′-GAACTTACAGGCGGGCTGGGT-3′ Ach5FtsZ-R: 5′-CGCCGTCTTCAGGGCACTTTCA-3′	62.0 62.1	369	(Hoekema et al. 1983)

Description of Genetic makeup and target regions for designing PCR primers of various disarmed *A. tumefaciens* strains used in the study.

A typical PCR reaction for individual primer sets contained about 50 ng DNA template, Taq buffer (10 mM Tris–HCl, pH 8.8, 50 mM KCl and 0.18% Nonidet P40) 1.5 mM MgCl_2_, 200 μM of each dNTPs, 1.5 units Taq DNA Polymerase (recombinant) (Fermentas UAB Lithuania), and 25 pM of each primer. The thermal profile for all the reactions included pre-PCR denaturation at 94°C for 3 minutes followed by 30 cycles of denaturing at 94°C for 20 seconds, annealing at 61°C for 20 seconds and extension at 72°C for 40 seconds, and a final extension at 72°C for 20 minutes. After PCR, products were electrophoresed on 1.5% agarose gel in TAE buffer (40 mM Tris-acetate, 1 mM EDTA pH 8.1), stained with ethidium bromide and visualized on a UV transilluminator.

For multiplex PCR, four sets of primers Ach5FtsZ-F/R, C58GlyA-F/R, pTiBo542-F/R and nptI-F/R were simultaneously added in concentrations of 25 pM each. The thermal profile for multiplexing was similar to the thermal profile of individual primer sets.

## Electronic supplementary material

Additional file 1: Figure S1: PCR amplification of different *Agrobacterium* strains using various primer sets developed in the study. (a) PCR results of Ach5FtsZ-F/R primer set with nine *Agrobacterium* strains. Lane 1–9 represents LBA4404, GV3101, C58C1, EHA101, EHA105, At 10, Ag57, Ag63 and R3 respectively. (b) PCR results of C58GlyA-F/R primer pair with nine *Agrobacterium* strains. Lane 1–9 represents GV3101, C58C1, EHA101, EHA105, LBA4404, At 10, Ag57, Ag63 and R3 respectively. (c) PCR results of pTiBo542-F/R primer set with nine *Agrobacterium* strains. Lane 1–9 represents EHA101, EHA105, LBA4404, GV3101, C58C1, At 10, Ag57, Ag63 and R3 respectively. (d) PCR results of nptI-F/R primer pair with nine *Agrobacterium* strains. Lane 1–9 represents EHA101, EHA105, LBA4404, GV3101, C58C1, At 10, Ag57, Ag63 and R3 respectively. In all panels first lane marked M represents O’RangeRuler™ 100 bp DNA Ladder (Fermentas Lithuania UAB). (PDF 71 KB)

Additional file 2: Figure S2: Multiplex PCR for specificity assessment of C58GlyA-F/R, Ach5FtsZ-F/R, pTiBo542-F/R and nptI-F/R primer pairs with *E. coli* strains DH5α and BL21. Multiplex PCR using combined primer sets to amplify total genomic DNA of EHA105 (used as positive control), DH5α and BL21 respectively (lanes 1–3). Only specific bands appeared in EHA105. M is O’RangeRuler™ 100 bp DNA Ladder (Fermentas Lithuania UAB). (PDF 61 KB)
