# Supplementary material for: Multiplex PCR assay for identification of commonly used disarmed Agrobacterium tumefaciens strains
Source: Springerplus. 2014 Jul 15;3:358. doi: 10.1186/2193-1801-3-358 (PMC4117855; doi:10.1186/2193-1801-3-358)
Supplement: Supplementary file 1 — Additional file 1: Figure S1: PCR amplification of different Agrobacterium strains using various primer sets developed in the study. (a) PCR results of Ach5FtsZ-F/R primer set with nine Agrobacterium strains. Lane 1–9 represents LBA4404, GV3101, C58C1, EHA101, EHA105, At 10, Ag57, Ag63 and R3 respectively. (b) PCR results of C58GlyA-F/R primer pair with nine Agrobacterium strains. Lane 1–9 represents GV3101, C58C1, EHA101, EHA105, LBA4404, At 10, Ag57, Ag63 and R3 respectively. (c) PCR results of pTiBo542-F/R primer set with nine Agrobacterium strains. Lane 1–9 represents EHA101, EHA105, LBA4404, GV3101, C58C1, At 10, Ag57, Ag63 and R3 respectively. (d) PCR results of nptI-F/R primer pair with nine Agrobacterium strains. Lane 1–9 represents EHA101, EHA105, LBA4404, GV3101, C58C1, At 10, Ag57, Ag63 and R3 respectively. In all panels first lane marked M represents O’RangeRuler™ 100 bp DNA Ladder (Fermentas Lithuania UAB). (PDF 71 KB) [file 40064_2014_1075_MOESM1_ESM.pdf]

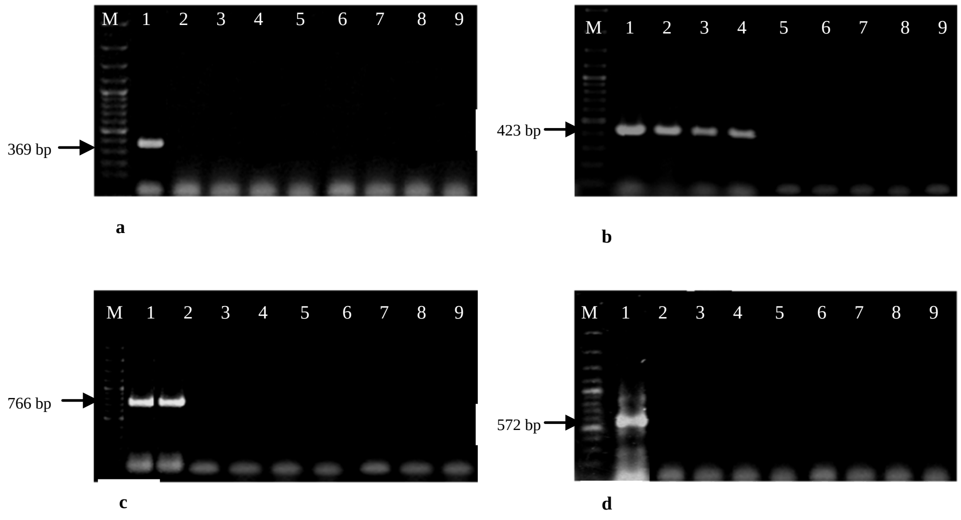

Additional Figure 1

PCR amplification of different *Agrobacterium* strains using various primer sets developed in the study. **(a)** PCR results of Ach5FtsZ-F/R primer set with nine *Agrobacterium* strains. Lane 1-9 represents LBA4404, GV3101, C58C1, EHA101, EHA 105, At 10, Ag57, Ag63 and R3 respectively. **(b)** PCR results of C58GlyA-F/R primer pair with nine *Agrobacterium* strains. Lane 1-9 represents GV3101, C58C1, EHA101, EHA 105, LBA4404, At 10, Ag57, Ag63 and R3 respectively. **(c)** PCR results of pTiBo542-F/R primer set with nine *Agrobacterium* strains. Lane 1-9 represents EHA101, EHA 105, LBA4404, GV3101, C58C1, At 10, Ag57, Ag63 and R3 respectively. **(d)** PCR results of nptI-F/R primer pair with nine *Agrobacterium* strains. Lane 1-9 represents EHA101, EHA 105, LBA4404, GV3101, C58C1, At 10, Ag57, Ag63 and R3 respectively. In all panels first lane marked M represents O’RangeRuler™ 100 bp DNA Ladder (Fermentas Lithuania UAB).
