# Supplementary material for: Multiplex PCR assay for identification of commonly used disarmed Agrobacterium tumefaciens strains
Source: Springerplus. 2014 Jul 15;3:358. doi: 10.1186/2193-1801-3-358 (PMC4117855; doi:10.1186/2193-1801-3-358)
Supplement: Supplementary file 2 — Additional file 2: Figure S2: Multiplex PCR for specificity assessment of C58GlyA-F/R, Ach5FtsZ-F/R, pTiBo542-F/R and nptI-F/R primer pairs with E. coli strains DH5α and BL21. Multiplex PCR using combined primer sets to amplify total genomic DNA of EHA105 (used as positive control), DH5α and BL21 respectively (lanes 1–3). Only specific bands appeared in EHA105. M is O’RangeRuler™ 100 bp DNA Ladder (Fermentas Lithuania UAB). (PDF 61 KB) [file 40064_2014_1075_MOESM2_ESM.pdf]

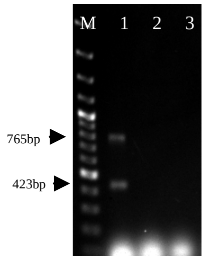

Additional Figure 2

Multiplex PCR for specificity assessment of C58GlyA-F/R, Ach5FtsZ-F/R, pTiBo542-F/R and nptI-F/R primer pairs with *E. coli* strains DH5α and BL21. Multiplex PCR using combined primer sets to amplify total genomic DNA of EHA105 (used as positive control), DH5α and BL21 respectively (lanes 1-3). Only specific bands appeared in EHA105. M is O’RangeRuler™ 100 bp DNA Ladder (Fermentas Lithuania UAB).
